# Non-TAL Effectors From *Xanthomonas oryzae* pv. *oryzae* Suppress Peptidoglycan-Triggered MAPK Activation in Rice

**DOI:** 10.3389/fpls.2018.01857

**Published:** 2018-12-12

**Authors:** Juying Long, Congfeng Song, Fang Yan, Junhui Zhou, Huanbin Zhou, Bing Yang

**Affiliations:** ^1^Key Laboratory of Monitoring and Management of Plant Diseases and Insects, Ministry of Education, Nanjing Agricultural University, Nanjing, China; ^2^Department of Genetics, Development, and Cell Biology, Iowa State University, Ames, IA, United States; ^3^Institute of Plant Protection, Chinese Academy of Agricultural Sciences, Beijing, China; ^4^Division of Plant Sciences, University of Missouri, Columbia, MO, United States; ^5^Donald Danforth Plant Science Center, St. Louis, MO, United States

**Keywords:** *Xanthomonas*, type III effector, TAL effector, non-TAL effector, MAPK, immunity, rice

## Abstract

*Xanthomonas oryzae* pv. *oryzae*, the causal pathogen of bacterial blight of rice, depends on its type III secretion system and associated effector proteins to grow and colonize the vascular tissues of rice plants. The type III effectors include a family of closely related transcription activator-like (TAL) effectors and the rest of diverse effectors, so-called non-TAL effectors. Our understanding of non-TAL effectors for pathogenesis in rice blight is still limited. Here we report a feasible method to rapidly detect the activation of mitogen-activated protein kinase pathway in rice mesophyll protoplasts by the *X*. *oryzae* pv. *oryzae* derived peptidoglycan and screen for virulent effectors that can suppress the pathogen-associated molecular pattern triggered immunity (PTI) response. Amongst 17 non-TAL effectors transiently expressed in rice cells, we found that three effectors (XopZ, XopN, and XopV) were able to suppress the peptidoglycan-triggered MAPK activation. The triple mutant of the *X. oryzae* pv. *oryzae* strain PXO99^A^ lacking *XopZ*, *XopN*, and *XopV* showed additively reduced virulence. Adding back either of genes restored the virulence of the triple mutant. Our results demonstrate the collective and redundant ability of defense suppression by non-TAL effectors in causing bacterial blight of rice.

## Introduction

Plants ward off the infection of microbial pathogens through two major layers of defense, namely pathogen-associated molecular pattern (PAMP) triggered immunity (PTI) and effector triggered immunity (ETI) (Boller and Felix, 2009). PTI is initiated upon perception of PAMPs by the pattern recognition receptors (PRRs) at the cell surface and subsequently activation of a cascade of gene expression and complex signaling pathways, leading to a basal disease resistance in plants. The PAMPs include lipopolysaccharide (LPS), peptidoglycan (PGN), flagellin, chitin, elongation factor Tu, glucan and even DNA fragments of bacterial and fungal origins. Bacterial and fungal pathogens can also use a suite of effector proteins to suppress the PTI to enable microbial growth and disease symptom development. The effectors are virulence factors *per se*. However, they could be recognized by host resistance genes or gene products, leading to stronger burst of host defense responses including the hallmark character of hypersensitive reaction (HR), a process of rapid, localized cell death, to limit the microbial proliferation, the second layer of host immunity (ETI) (Jones and Dangl, 2006).

Bacterial blight of rice, inflicted by the pathogenic *Xanthomonas oryzae* pv. *oryzae* (*Xoo*), causes great losses of rice production in south Asian and west African countries (Mew et al., 1993). *Xoo* cells enter rice leaves through hydathodes and wounds, followed by their colonization and spread in vascular tissues of leaves and sheaths, and eventually cause leaf blight characteristic of gray to white opaque necrotic lesion in leaf and stem of rice plants (Niño-Liu et al., 2006). Disease control based on genetic resistance remains the only effective way in the field (Mew, 1987).

At the molecular level, the blight is the outcome of interactions between host rice and pathogen *Xoo*. For example, the apoplast-dwelling pathogen uses its type III secretion system (T3SS) to translocate the bacterial effector proteins into host rice cells to manipulate the host transcriptional and physiological processes, rendering host more susceptible to bacterial growth and symptom progression. In this case, each strain of *Xoo* contains about 35 type III effectors that can be divided into two groups: TALEs (transcription activator-like effectors) and non-TALEs. The interaction of TALEs and host genes can be characterized as protein/DNA interaction, i.e., TALEs, once internalized into nuclei of host cells, recognize the promoters and transcriptionally activate the disease susceptibility genes, inducing a state of disease. In addition, a group of TALE variants (e.g., so-called iTALEs or truncTALEs, due to C-terminal truncations of typical TALEs) involve ETI suppression of XA1 mediated resistance against most, if not any, full-length TALEs, which could be characterized as protein/protein interaction (Ji et al., 2016). On the other hand, the non-TALEs include a suite (ca.18-23) of structurally diverse type III effectors. The functions of some non-TALEs have been characterized as virulence factors by suppressing PTI in bacterial blight of rice (Song and Yang, 2010; Akimoto-Tomiyama et al., 2012; Yamaguchi et al., 2013a,b; Zhao et al., 2013; Ishikawa et al., 2014; Wang et al., 2016; Qin et al., 2018). Overall, the studies for the molecular interaction have been largely focused on the TALE biology and major breakthrough have been made in the last decades. However, our understanding of type III effectors beyond TALEs (so called non-TALEs) is limited.

In present study, we established a protoplast system to study the MAPK activation as one of PTI processes in rice and screen type III effectors for PTI suppressors. The PTI process involves the MAPK (mitogen-activated protein kinase) activation in response to peptidoglycan (PGN), a common PAMP extracted from *Xoo*. We identified three Xop (*Xanthomonas* outer protein) effectors that were able to suppress the MAPK activation and revealed their redundant role in virulence in *Xoo* infection process and lesion formation.

## Materials and Methods

### Plant, Bacterial Strains, and DNA Manipulations

The wild-type *Arabidopsis thaliana* Col-0 was used for isolation of leaf mesophyll protoplasts from 3–4-week-old plants. The *japonica* rice (*Oryza sativa*) cultivar Kitaake was used for isolation of protoplasts from young seedlings and 5–6 weeks old plants for disease assay. Strains of *Escherichia coli* and *Xoo* used in this study are listed in Table 1. Bacterial culture and DNA manipulation were performed with standard techniques (Ausubel et al., 1998). *Xoo* was grown in either nutrient broth (NA) (BD, Difco) or tryptone sucrose medium (TS) (tryptone, 10 g; sucrose, 10 g; glutamic acid, 1 g; Difco Bacto agar, if solid, 15 g per liter) at 28°C. Plasmids were transferred into *E. coli* or *Xoo* through electroporation. Antibiotics used in this study were ampicillin (100 μg/ml), cephalexin (10 μg/ml), kanamycin (50 μg/ml), and spectinomycin (100 μg/ml).

**Table 1:** Bacterial strains and plasmids used in this study.

Designation	Genotypes or related characteristics	Source or reference
**Plasmids**		
pUC:35S	35S-MCS-NOS cassette in pUC19, ampicillin resistance	This study
pOsMAP1	*OsMAP1* in pUC:35S, ampicillin resistance	This study
pOsMAP5	*OsMAP5* in pUC:35S, ampicillin resistance	This study
pHM1	Broad host range, resistance to spectinomycin, *cos* site	Hopkins et al., 1992
pKMS1	A suicide vector for marker exchange mutagenesis	Zou et al., 2011
pXopZ	*XopZ* genomic clone in pHM1	Song and Yang, 2010
pXopN	*XopN* genomic clone in pHM1	This study
pXopV	*XopV* genomic clone in pHM1	This study
p35S:Xop-HA	pUC:35S plasmids individully expressing each of 17 non-TALE effector genes as indicated in text	
***Escherichia coli***		
DH5α	F′ *recA, ϕ80dlacZ*, *ΔM15*	Stratagene, CA
XL1-Blue MRF’	F′ *proAB*, *lacI^q^Z*, *ΔM15,* Tn10 (Tet^r^)	Stratagene, CA
C2110	Nal^r^,Rf^r^ (*polA1*, *rha*, *his*)	Leong et al., 1982
BL21 Star (DE3)pLysS	F^-^ *^ompThsdS^*B (rB^-^mB^-^) *gal dcm rne131* (DE3) pLysS (Cam^R^)	Thermo Fisher Scientific, MA
***Xanthomonas oryzae* pv. *oryzae***		
PXO99^A^	Philippine race 6, azacytidine resistant clone of PXO99	Hopkins et al., 1992
ΔXopZ	*XopZ* knockout of PXO99^A^	Song and Yang, 2010
ΔXopN	*XopN* knockout of PXO99^A^	This study
ΔXopV	*XopV* knockout of PXO99^A^	This study
ΔXopZ;N;V	Triple knockout of *XopZ*, *XopN* and *XopV* in PXO99^A^	This study
ΔXopZ;N;V(*XopZ*)	ΔXopZ;N;V transformed with plasmid borne *XopZ*	This study


### Plasmid Construction

The plasmids for expression of rice *MPK1* (Os06g06090) and *MPK5* (Os03g17700) were constructed by cloning their cDNA-derived PCR (polymerase chain reaction) amplicons into pUC:35S. pUC:35S was a pUC19 derived vector containing the 35S promoter, Nos terminator and multiple cloning sites (MCS) between. The PCR fragment of each *MPK* was cloned into pUC:35S between the *Bam*HI and *Hin*dIII restriction sites of MCS and in frame at its 3′ end with a FLAG-coding sequence. pHBT-DMEKK1-FLAG expressing the Arabidopsis *ΔMEKK1* was kindly provided by Ping He. All non-TAL effector genes were PCR-amplified with gene-specific primers and genomic DNA of PXO99^A^ using the Phusion high-fidelity DNA polymerase (New England Biolabs). The amplicons were individually cloned at the appropriate restriction sites into an expression vector a modified pUC:35S containing the 35S promoter and in fused with a sequence encoding an HA epitope-tag at the C terminus. Construct to express the *Xoo* flagellin was made through PCR-amplification of *fliC* coding region with genomic DNA of PXO99^A^ and cloning into the *E*. *coli* expression vector pHTb at *Bam*HI and *Hin*dIII. The recombinant protein flagellin N-terminally fused with 6xHIS tag was produced in BL21 Star (DE3)pLysS (Thermo Fisher Scientific) and purified with Ni-NTA Agarose (QIAGEN) following the manuals of both manufacturers. The primer sequences for PCR-amplification are provided in Supplementary Table S1.

### Protoplast Preparation and PEG-Mediated Transfection

The *Arabidopsis* mesophyll protoplasts were isolated from rosette leaves as previously described (Yoo et al., 2007). Rice protoplast preparation and transfection were carried out as reported before (Jiang et al., 2013). Briefly, rice seed was surface-sterilized with 50% bleach, germinated and grown on 1/2 MS medium + B5 Vitamins containing 1.5% sucrose and 0.6% agar in ice cream cone cups in growth chamber at 30°C and with 12 h lighting. Leaves and leaf stems of 8–10 days old seedlings were used for protoplast isolation and PEG-mediated transfection. The protoplasts were harvested at the time points post-transfection as specified in test for further analysis. For immunoblot detection of endogenous MAPK activation and pull-down assay, an aliquot of 0.8 ml of protoplasts at a density of 2 × 10^6^/ml was transfected with 30 μg of each plasmid DNA (*OsMAPK1*, *OsMAPK5*, *OsMAPK6*, *ΔMEKK1* and/or *Xop* genes). Each experiment was repeated at least three times.

### Peptidoglycan (PGN) Extraction and Elicitor Treatment

Isolation and purification of PGN were carried out from the Xoo strain PXO99^A^ as described previously (Barnard and Holt, 1985). Briefly, the bacterial cells were boiled in 100 volumes of 5% SDS for 30 min. After cooling down to room temperature, the SDS-insoluble material was collected by centrifuging at 30,000 g at 4°C for 30 min. The pellet was treated with trypsin digestion to remove protein contamination and dialyzed extensively against distilled water.

*Arabidopsis* and rice protoplasts were treated with flg22 (Sigma-Aldrich) at a final concentration of 1 μM. Chitin (Sigma-Aldrich) was applied to rice protoplasts at a final concentration of 10 μM. Before flg22 or chitin treatment, rice and *Arabidopsis* protoplasts were isolated and incubated for about 12 h in W5 solution under dark, then incubated under light for 2 h in fresh W5 solution. After treatment (e.g., 10 min), protoplasts were harvested for immunoblot analysis. For PGN treatment, protoplasts were collected after 12 h post-transfection of effector gene constructs and resuspended in 100 μl of W5 solution. After recovered under light for 2 h in the 30°C growth chamber, protoplasts were treated with 5 mg/ml PGN for 15–60 min and then harvested for immunoblot analysis.

### Pull-Down Assay

Total proteins were extracted from protoplasts with 0.5 ml of lysis buffer that is composed of 50 mM Tris-HCl, pH 7.5, 100 mM NaCl, 10% glycerol, 0.1% NP-40, 0.5 mM DTT, 1 mM PMSF and protease inhibitor cocktail (Sigma-Aldrich). Cell debris was pelleted at 20,000 *g* for 15 min at 4°C. The supernatants were incubated with 30 μl of Anti-FLAG M2 Magnetic Bead (Sigma-Aldrich) for at least 1 h at 4°C with gentle shaking, the beads were then washed three times with lysis buffer. An aliquot of 2X SDS loading buffer (25 μl) was added to the beads, and then heated at 95°C for 5 min. 10 μl of each sample was loaded on the protein gel for immunoblot analysis as described below.

### Immunoblot Analysis

Proteins were separated through electrophoresis in 12% SDS-PAGE gel and electro-transferred to Hybond-P PVDF membranes (Amersham Biosciences) for blotting. Immunodetection was carried out using standard methods. The anti-HA, anti-FLAG (Santa Cruz Biotechnology) and anti-Phospho-p44/42 MAPK (Erk1/2) (Thr202/Tyr204) antibodies (Cell Signaling Technology) were used at a dilution of 1:1,000. HRP-conjugated secondary antibody (Sigma-Aldrich) was used at a dilution of 1:20,000. Blots were visualized with SuperSignal West Pico Chemiluminescent Substrates (Thermo Scientific) following the manufacturer’s instructions.

### Generation of Individual and Triple Mutant for *XopN*, *XopV,* and *XopZ*

The suicide vector pKMS1 was used to individually and sequentially delete the three Xop genes (*XopN*, *XopV,* and *XopZ*) for a triple mutant using a method as described (Zou et al., 2011). Specifically, two pairs of gene-specific primers (sequences provided in Supplementary Table S1) were designed to match two separate regions of each gene based on the PXO99^A^ genome sequence (NCBI accession, CP000967).

The PCR was performed to amplify the upstream and downstream regions of each locus by using the PXO99^A^ genomic DNA as template. The two PCR amplicons for each gene deletion were fused by overlap PCR and cloned into the MCS (multiple cloning site) of pKMS1 and confirmed by sequencing for accuracy of sequence. The mutagenesis was performed on PXO99^A^ targeting individual *Xop* genes. Plasmid DNA was electroporated into competent cells of PXO99^A^ and transformants were plated on the kanamycin NA lacking sucrose. Single colonies were transferred to nutrient broth medium lacking sucrose and incubated with shaking for 12 h at 28°C. Bacterial cells were then plated on NA containing sucrose. Sucrose tolerant colonies were duplicated on NA and kanamycin-containing NA plates. The kanamycin-sensitive colonies were screened for gene deletions by PCR with gene deletion-specific primers. The mutants were used for sequential deletion through the second and third rounds of mutagenesis for triple mutant using the similar approach.

### Disease Assay

The rice plants were grown in growth chamber containment with temperature of 28°C and relative humility of 75% and photoperiod of 12 h in light/dark. Fully expanded leaves of 2-month-old rice plants (*n* = 5–10) were used for leaf tip-clipping inoculation with the bacterial concentration at 0.5 OD_600_ (approximately 5.0 × 10^7^). Lesion lengths were measured 12 days post inoculation (DPI) from leaf tip to the diseased edge of lesions. One-way analysis of variance statistical analyses was performed on all measurements. The Tukey’s honest significant difference test was used for post analysis of variance pair-wise tests for significance, set at 5% (*p* < 0.05).

## Results

### Establishment of a Method to Rapidly Study PAMP-Triggered Immunity in Rice Mesophyll Protoplasts

**FIGURE 1:**
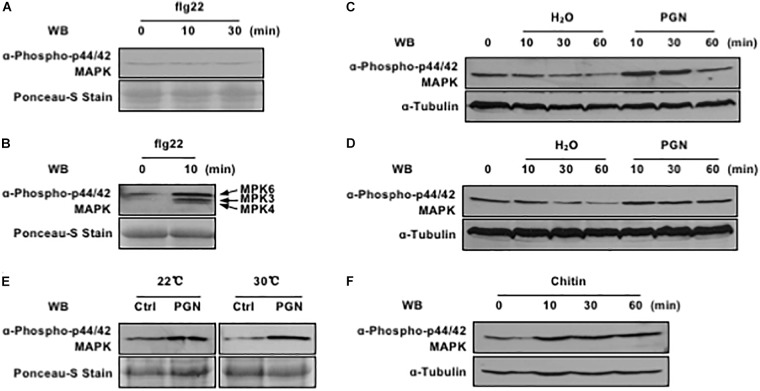
Pathogen-associated molecular pattern (PAMPs) activate MAPK cascade in rice protoplasts. **(A)** Rice protoplasts do not respond to flg22 treatment. Rice protoplasts were treated with 1 μM flg22, MAPK activation (top) and loading control (bottom) are shown. **(B)** flg22 activates endogenous MAPK cascade in *Arabidopsis* protoplasts. *Arabidopsis* protoplasts were treated with 1 μM flg22, MAPK activation (top) and loading control (bottom) are shown. **(C)** Endogenous MAPK activation is induced by PGN under the lights. Rice protoplasts were treated with or without 5 mg/ml PGN, MAPK activation (top) and loading control (bottom) are shown. **(D)** PGN has weak effect on endogenous MAPK activation in the dark. Rice protoplasts were treated with or without 5 mg/ml PGN, MAPK activation (top) and loading control (bottom) are shown. **(E)** Endogenous MAPK cascade is activated at different temperature under the lights. Rice protoplasts were treated with or without 5 mg/ml PGN under the lights for 10 min, MAPK activation (top) and loading control (bottom) are shown. **(F)** Chitin activates endogenous MAPK cascade in rice protoplasts. Rice protoplasts were treated with 10 μM chitin, MAPK activation (top) and loading control (bottom) are shown. The data shown here are one representative of three independent experiments.

A number of non-TAL effectors of *Xanthomonas* genus were identified in suppressing flg22-induced signaling pathway and inhibiting early defense gene expression in the dicot model plant *A. thaliana* (Popov et al., 2016; Wang et al., 2016). However, little is known about its real functions in the natural host species, like rice. Therefore, we sought to set up a simple and quick system for PAMP signaling studies and screening for virulent effectors in the rice mesophyll protoplasts by immunoblotting with p44/42 MAP kinase antibody, which specifically recognizes phosphorylated forms of MAPK kinases. When the widely used flg22 peptide was first applied in the rice protoplasts which were recovered in the dark at room temperature, no elevated OsMAPK activation was observed at the early time points (Figure 1A). By contrast, flg22 perception triggered strong MAPK activation in *Arabidopsis* protoplasts within 10 min under the same conditions (Figure 1B). Furthermore, when flagellin of *Xoo* was ectopically expressed in *E. coli*, purified and used to treat rice protoplasts, no OsMAPK activation was detected either, which is consistent with the notion that *Xoo* evades the flagellin detection system in rice (Wang et al., 2015). Therefore, we assume that the *Xoo* flagellin and the *Pseudomonas syringae* flg22 epitope may not be the conserved PAMPs to induce PTI responses in rice.

In this regard, peptidoglycan (PGN), another type of the well-known PAMPs, was extracted from the PXO99^A^ and further tested for its capability of triggering OsMAPK activation with and without lights at room temperature. As shown in Figure 1, PGN induced OsMAPK phosphorylation more obviously under lights (Figure 1C) compared to that in the dark (Figure 1D); the enhanced level was maintained at 30 min and restored to the normal level at 60 min with PGN treatment. Next, we tested if high temperature would affect the PGN-induced OsMAPK phosphorylation level given that the optimal growth temperature for rice is near 30°C. The rice protoplasts were recovered and treated with PGN at different temperatures (20 and 30°C), we found that rapid induction of OsMAPK phosphorylation occurred within 10 min at both temperatures and it was more consistent at 30°C than 20°C (Figure 1E). Thus, we carried out all downstream experiments with the rice protoplast system under lights and at 30°C. In addition, chitin, a typical fungal PAMP that trigger various defense responses in both monocots and dicots, was tested in our system as well. The results revealed that it triggered rapid activation of OsMAPK cascade as PGN did (Figure 1F). Taken together, these data indicate an establishment of a fast and sensitive system for test of PTI in rice protoplasts, which is suitable for studies on PTI-related molecules derived from both bacterial and fungal pathogens.

### PGN-Triggered PTI Pathway in Rice

**FIGURE 2:**
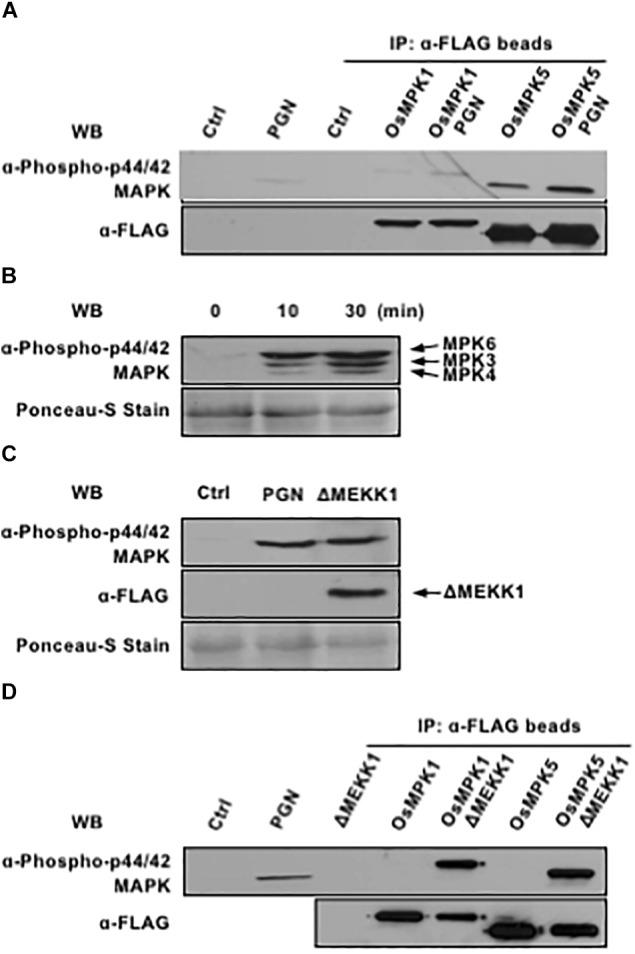
OsMPK1 and OsMPK5 mediate PGN-induced MAPK activation in rice protoplasts. **(A)** OsMPK1 and OsMPK5 are activated by PGN in rice protoplasts. OsMPK1 or OsMPK5 were expressed in rice protoplasts overnight and immuno-precipitated with anti-FLAG-beads after 5 mg/ml PGN treatment for 10 min. MAPK activation (top) and MAPK expression (bottom) are shown. **(B)** PGN extracted from *Xoo* activates endogenous MAPK cascade in *Arabidopsis* protoplasts. *Arabidopsis* protoplasts were treated with 5 mg/ml PGN. MAPK activity (top) and loading control (bottom) are shown. **(C)** Constitutively active AtMEKK1 activates endogenous MAPK cascade in rice protoplasts. Rice protoplasts were transfected with construct expressing constitutively active MAPKKK of *Arabidopsis*. MAPK activation (top), MAPKKK expression (middle) and loading control (bottom) are shown. **(D)** Constitutively active AtMEKK1 activates OsMPK1 and OsMPK5 in rice protoplasts. *OsMPK1* or *OsMPK5* were co-expressed with constitutively active *AtMEKK1* in rice protoplasts overnight, and then immuno-precipitated with anti-FLAG-beads. MAPK activation (top) and MAPK expression (bottom) are shown. The data shown here are representative of five independent experiments.

Chitin-induced MAPK activation is regulated by common homologous elements in both rice and *Arabidopsis*, which involves pattern-recognition receptors (PRRs), receptor-like cytoplasmic kinases (RLCKs) and three sequentially activated mitogen-activated protein kinases (MAPKs) (Kawasaki et al., 2017; Yamada et al., 2017). Early studies show that OsLYP4/6 and OsRLCKs play important roles in PGN signaling, but little is known about OsMAPK functioning downstream of PAMP perception (Liu et al., 2012; Ao et al., 2014; Li et al., 2017). To address this question, we cloned three rice homologous genes (*OsMAPK1*, *OsMAPK5,* and *OsMAPK6*) of *Arabidopsis MPK3*, *MPK4,* and *MPK6* which are important in the flg22-dependent phosphorylation pathway related to PTI defense response, expressed individually in rice protoplasts followed by PGN treatment, immuno-precipitated with anti-FLAG M2 magnetic beads, and tested for activation by immunoblotting with p44/42 MAP kinase antibody. The results indicated that both OsMAPK1 and OsMAPK5 were activated after PGN application (Figure 2A). The involvement of OsMAPK6 cannot be determined, however, resulting from its poor expression.

Meanwhile, we examined whether PGN extracted from the *Xoo* was able to activate MAPKs in *Arabidopsis* protoplasts as well. Treatment of protoplasts with PGN induced rapid and strong MAPK activation within 10 min (Figure 2B), suggesting PGN is a conserved PAMP that could be recognized in both rice and *Arabidopsis*. Therefore, ΔMEKK1, a constitutively active catalytic domain (326–592) of MEKK1 that is known to activate MPK3 and MPK6 in *Arabidopsis* (Asai et al., 2002), in plasmid pHBT-DMEKK1-FLAG, was tested for activating OsMAPKs in rice protoplasts. The results showed that transient expression of the *Arabidopsis*-derived ΔMEKK1 resulted in OsMAPK activation to an extent as PGN did (Figure 2C). The FLAG-tagged OsMAPKs were further co-expressed individually with ΔMEKK1 in rice protoplasts, immunoprecipitated and tested for activation. As shown in Figure 2D, both OsMAPK1 and OsMAPK5 were highly phosphorylated in presence of ΔMEKK1. In light of these findings, we assume that PGN-triggered activation of MAPK cascades is conserved in rice and *Arabidopsis*.

### Identification of the Virulent Non-TAL Effectors of *Xoo* Related to PGN-Triggered PTI

Type III secreted effectors interfere with plant cellular pathways to benefit the pathogen and promote bacterial multiplication during infection. Of which, targeting MAPK cascades has been found to be a common strategy for a wide range of bacterial pathogens (Bi and Zhou, 2017). However, compared to Hop effectors from *Pseudomonas* genus that have been intensively studied, most of non-TAL effectors from *Xoo* have not been well characterized. Therefore, we sought to identify the virulent effectors of *Xoo* that have potential to suppress OsMAPK cascades in rice.

**FIGURE 3:**
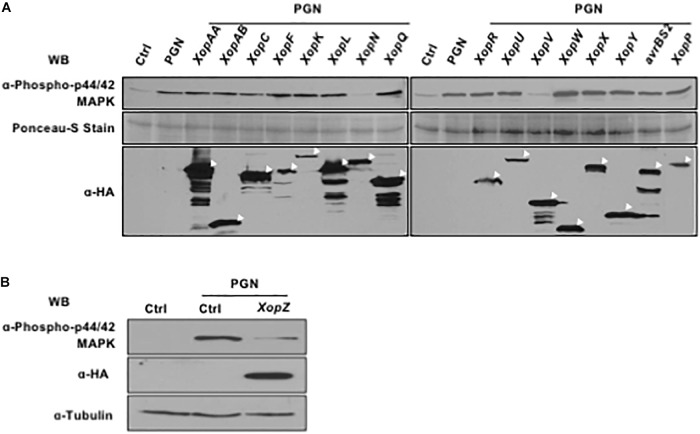
Non-TAL effectors of *Xoo* suppress endogenous MAPK cascades induced by PGN. **(A,B)** Rice protoplasts were transfected with constructs individually expressing Non-TAL effectors overnight and then treated with 5 mg/ml PGN for 15 min. MAPK activation (top), non-TAL effector expression (bottom, indicated with white arrow heads) and loading control (bottom) are shown. All experiments were repeated three times with the similar results.

There are 18 non-TAL effector-encoding genes, including 2 identical copies of *XopZ*, annotated in the genome of the *Xoo* strain PXO99^A^ (Song and Yang, 2010). Therefore, we cloned a total of 17 *Xop* genes and expressed the HA-tagged fusion proteins individually in rice protoplasts followed by PGN treatment. Western blot analysis with p44/42 MAP kinase antibody clearly showed that XopN and XopV inhibited the PGN-induced phosphorylation of OsMAPKs (Figure 3A). XopZ, a virulent effector we identified previously through mutagenesis of the strain PXO99^A^, was also capable of inhibiting the activation (Figure 3B). Expression of all Xop effectors were verified by immunoblotting with anti-HA antibody (Figures 3A,B, the lower panels). Thus, expression of three Xop effectors from PXO99^A^ in rice protoplasts results in compromised OsMAPK activation induced by PGN, highlighting their putative virulence functions during pathogenesis.

### The Virulence Contribution of *XopZ*, *XopN,* and *XopV*

**FIGURE 4:**
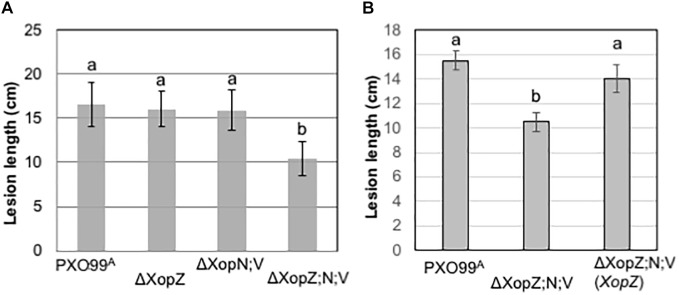
Non-TAL effectors XopZ, XopV, and XopN function in the pathogenicity of *Xoo* PXO99^A^ in rice. **(A,B)** Measurements of lesion lengths caused by PXO99^A^ and its derivatives mutants as indicated below each column. The measurements each were obtained from 10 leaves of five rice plants of Kitaake (two leaves per plant). Values with the same lowercase letters above columns do not differ significantly at the <0.5 level using Tukey statistics and ANOVA analysis. Bars indicate standard deviation (SD).

Since three Xop effectors in PXO99^A^ are individually capable of suppressing MAPK activation induced by PGN in rice protoplasts and MAPK signaling is known to play an important role in PTI in rice, we resonate that those Xop effectors are important virulence factors in blight disease. We next tested the individual and collective role of virulence played by the three Xop effectors in pathogenesis with PXO99^A^. Single, double and triple gene mutants were generated by deleting, individually and in combination, *XopN*, *XopV* and two copies of *XopZ* in PXO99^A^. All the mutants grew in nutrient medium normally without obvious growth defect. The disease assay through leaf tip-clipping inoculation in *japonica* rice variety Kitaake revealed no obvious virulence reduction by mutants of individual *XopN* and *XopV* (Supplementary Figure S1), like in IR24, an *indica* rice variety in a prior study (Song and Yang, 2010). The double mutant of *XopN* and *XopV* also caused lesion lengths similar to the wild type (Figure 4A). In contrast to the prior disease assay in IR24, *XopZ* single mutant lacking two *XopZ* loci did not show obvious different lesion length from PXO99^A^ in Kitaake (Figure 4A). However, a significant decrease in lesion length with the triple mutation was observed (Figure 4A). The reduction in virulence in the triple mutant was restored by introduction of *XopZ* (Figure 4B), *XopN* and *XopV* (Supplementary Figure S1) in term of lesion lengths in Kitaake. The results indicate that *XopZ*, *XopN*, and *XopV* collectively contribute strain virulence in rice Kitaake.

## Discussion

In our study, we established a rice protoplast system and used it to detect the early and quick activation of MAPK after treatment with PGN derived from *Xoo*. The system enabled us to identify individual XopN, XopV, and XopZ that were capable of interfering MAPK signaling pathway through transiently expressing each protein from a complement of 17 non-TALEs from PXO99^A^. Plant species including rice respond to microbial infection by activating multiple defense mechanisms. MAPK signaling pathway represents such an important component in coordinating the early detection and quick defense response to invading plant pathogens (Pedley and Martin, 2005). MAPK cascades are used by eukaryotic organisms to transduce signals upon perception of extracellular molecules derived from bacterial and fungal pathogens (Bi and Zhou, 2017). The protoplast system can be used as a simple and reliable system to study some aspects of host cellular response to pathogen stimuli. MAPK activation and its mediated host defense responses against bacterial and fungal diseases have been demonstrated in rice (Reyna and Yang, 2006; Yang et al., 2015; Jalmi and Sinha, 2016; Ma et al., 2017). The use of protoplasts enables to synchronize the whole collection of cells to respond to the stimulus (PGN in our study), resulting in quick and uniform activation of MAPK activation. In contrast, it is challenging to achieve this in planta where tissues contain multiple layers of cells and a waxy structure on surface. Furthermore, expression of individual effector protein from a suite of type III effectors in protoplasts enables us effectively to tease out the consequence of other effectors, while in a natural phytopathogen systems, including bacterial blight of rice, pathogen secretes a repertoire of effector proteins into host cells to collectively benefit the infection and disease development (Galán et al., 2014; Büttner, 2016). It is worthy to note that the protoplast system may have a limitation in discovery of effectors that act on the host preinvasive immunity, such as stomatal defense, which is the first layer of barrier that pathogen should overcome to get entry of the host extracellular spaces (Melotto et al., 2006, 2017).

Bacterial blight of rice is one of the most important crop diseases and a model for studying host/microbe interaction (Zhang and Wang, 2013). The interaction is bridged by a T3SS and exerted by factors from both pathogen *Xoo* and its host rice. The pathogenesis determinants associated with T3SS include a large family of TALEs and about 20 non-TALE proteins in individual *Xoo* strains^1^. Several non-TAL type III effectors (e.g., XopK, XopP, XopR, XopY, XopZ, and XopAA) have been found to suppress PTI in rice or the heterologous systems (Song and Yang, 2010; Akimoto-Tomiyama et al., 2012; Yamaguchi et al., 2013a,b; Ishikawa et al., 2014; Wang et al., 2016; Qin et al., 2018). For example, XopR from *Xoo*, when conditionally expressed in Arabidopsis, was capable of enhancing bacterial growth of a T3SS defective *hrcC* mutant of *X. campestris* pv. *campestris*, and also capable of suppressing the induction of defense genes by this mutant and callose deposition induced by the flg22 peptide of flagella (Akimoto-Tomiyama et al., 2012). In another study, XopR has been found to physically interact with the rice BIK1 protein, a receptor-like cytoplasmic kinase (RLCK) and component of immune receptor complex, and it again, when expressed in Arabidopsis, suppresses PAMP-triggered stomatal closure (Wang et al., 2016). It has also been found that some non-TAL effectors suppress the upstream components of MAPK pathway, i.e., OsRLCK185 by XopY (or Xoo1488) and OsBAK1 by XopAA (or Xop875), and OsSERK1 by XopK (Yamaguchi et al., 2013a,b; Qin et al., 2018). Finally, XopP has been found to interact with the rice E3 ubiquitin ligase OsPUB44 and inhibit its ligase activity, thus suppressing immunity to *Xoo* in rice (Ishikawa et al., 2014). However, the molecular or biochemical mechanism in interfering with host immunity by other effectors remains unknown. By using MAPK activation in response to PGN as a readout coupled with expression of individual 17 non-TALEs, we found that three of them could suppress the MAPK activation, suggesting MAPK signal pathway as the target of those three effectors for virulence in blight disease. However, what exactly the interference in the cascade of MAPK (e.g., MAP kinases or upstream MAPKK or MAPKKK) by each of three Xop effectors remains to be determined.

In the model *Xoo* strain PXO99^A^, 18 type III effectors were identified as non-TALEs with XopZ as a virulence factor through systematic mutagenesis. XopZ was found to suppress the callose deposit in *Nicotiana benthamiana* when ectopically expressed. The *XopZ* knockout mutant of PXO99^A^ showed reduced virulence when it was inoculated in the *indica* rice IR24 (Song and Yang, 2010). However, when the same mutant was assessed in the *japonica* rice Kitaake, no obvious virulence reduction was observed. This might be due to the host genetic context or genetic interaction of both host and pathogen. Similar phenomenon has been observed in XopK from PXO99^A^ (Kitaake vs. IR24) (Qin et al., 2018) and XopN of KXO85 (Cheong et al., 2013). Similarly, single gene knockouts for other two non-TAL effectors XopN and XopV did not show obvious virulence reduction either, which is consistent with that in the prior study (Song and Yang, 2010). Indeed, the triple mutant of *XopN*, *XopV,* and *XopZ* showed significant virulence reduction compared to their single knockout and parental strains, and the phenotype could be restored by introduction of single *Xop* gene, suggesting an involvement of MAPK activation in immunity and functional redundancy of those three Xop effectors in MAPK suppression. Better understanding of how those virulence determinants in *Xoo* to manipulate host MAPK signaling pathways will enhance host disease resistance by delicately engineering MAPK cascade given the fact that MAPK involves multiple processes of biotic and abiotic stress tolerance (Meng and Zhang, 2013; Xu and Zhang, 2015).

## Author Contributions

JL, CS, FY, and JZ performed the experiments and analyzed the data. HZ and BY conceived, designed, and coordinated the research. All authors read and approved the final manuscript.

## Conflict of Interest Statement

The authors declare that the research was conducted in the absence of any commercial or financial relationships that could be construed as a potential conflict of interest.

## Footnotes

1 http://www.xanthomonas.org
