# Supplementary material for: Non-TAL Effectors From Xanthomonas oryzae pv. oryzae Suppress Peptidoglycan-Triggered MAPK Activation in Rice
Source: Front Plant Sci. 2018 Dec 12;9:1857. doi: 10.3389/fpls.2018.01857 (PMC6315156; doi:10.3389/fpls.2018.01857)
Supplement: Supplementary file 1 [file Data_Sheet_1.PDF]

Supplementary Table S1. **Primers in this study**

**Primer name**	**Sequence (5'- 3')**	**Usage**
OsMAP1-F	ATGGATCCAAATCCGAATCCGGCCATGG	Overexprssion of *OsMAP1* expression in rice protoplast
OsMAP1-R	ATAAGCTTCTGGTAATCAGGGTTGAACGCAAG	
OsMAP5-F	ATGGATCCAAGAGGAGGAGGGATTAGGGATGG	Overexprssion of *OsMAP5* expression in rice protoplast
OsMAP5-R	ATAAGCTTGTACCGGATGTTTGGGTTCATCTC	
AvrBs2-F	CCGGATCCAGGGTACCATGCGTATAGGTCCTCCGCAAAC	Overexprssion of *avrBs2* in rice protoplasts
AvrBs2-R	CCCTCGAGCTCCGGCTCGGTCTGGTTG	
XopC-F	GGGAATTCATGTGGCACGTTCAAGGTCATG	Overexprssion of *XopC* in rice protoplasts
XopC-R	CCCTCGAGTTTCCGGGCTTTCTCAACCAC	
XopF-F	GGGAATTCATGAAACTCTCCGGCGGTATC	Overexprssion of *XopF* in rice protoplasts
XopF-R	GCCCTCGAGTGCTCGCCCGCTTTGCCACT	
XopK-F	GGGAATTCATGCGCGTCCAGCGATCCACAGC	Overexprssion of *XopK* in rice protoplasts
XopK-R	CCCTCGAGGGTCGTGGACGCAGCAGCCTGTTC	
XopL-F	GGGAATTCATGAGGAAGGCGATGCGACGCGTC	Overexprssion of *XopL* in rice protoplasts
XopL-R	CCCTCGAGCGAAGGTTCCGAGGTTG	
XopN-F	CCGGATCCAGGGTACCAATACGCCCAGTTCCAGGCTG	Overexprssion of *XopN* in rice protoplasts
XopN-R	CCCTCGAGCGCCGGCGGCAGTGCCCGATCCT	
XopP-F	GGGAATTCATGTCACGCACATCGACATGAG	Overexprssion of *XopP* in rice protoplasts
XopP-R	CCCCTCGAGCTGTTGCCCACCAGCGCCAG	
XopQ-F	GGGAATTCATGCAGCCCACCGCAATCCGCTC	Overexprssion of *XopQ* in rice protoplasts
XopQ-R	CCGTCGACGCGCGCATGTTCCCCCTCGTC	
XopR-F	GGGAATTCATGCGCACGAATTTTCTTCCGCG	Overexprssion of *XopR* in rice protoplasts
XopR-R	CCGGATCCTCGGTAACCGTTCTCCATTGAG	
XopU-F	CAGGTACCGGGATCCGAATGAGGTGTCAGCCCATGACTC	Overexprssion of *XopU* in rice protoplasts
XopU-R	CCCTCGAGCGCGCGCCGACGCTGCCTGGCCTGCTT	
XopV-F	GGGAATTCATGAAAATCTCCGGCTCAGCGTC	Overexprssion of *XopV* in rice protoplasts
XopV-R	CCGAATCCTTCACCGTTAGGGTCAGAATG	
XopW-F	CCGGATCCAGGGTACCATGAAACCGAGCCACATCGGCAA	Overexprssion of *XopW* in rice protoplasts
XopW-R	CCCTCGAGAATTCGACTGCCGCTACTGGAG	
XopX-F	GGGAATTCATGCTGTGGCTGCGCCTGTTC	Overexprssion of *XopX* in rice protoplasts
XopX-R	CCCTCGAGATGCAGCGTCGAAGGACGGTC	
XopY-F	GGGAATTCATGCGCCCTGTCCAGCCCAATC	Overexprssion of *XopY* in rice protoplasts
XopY-R	CCCTCGAGCCGCCGGAAGACCGACTG	
XopZ-F	CCGGATCCACATGGGTCGCTTGCCTGCACCCG	Overexprssion of *XopZ* in rice protoplasts
XopZ-R	CCGGATCCCTCGAGCGGGACTGGCTCGTAGGGAATC	
XopAA-F	GGAGATCTCGGGTACCATGCAAATCAAAACCGCAAGCCA	Overexprssion of *XopAA* in rice protoplasts
XopAA-R	CCGGATCCCGACTGAGGCGCCGGCTGCA	
XopAB-F	GGGAATTCATGCCTCGCTCGCCAACTGCCT	Overexprssion of *XopAB* in rice protoplasts
XopAB-R	CCCTCGAGCACCAAGTCCTTGGCGTGTTTG	

XopN-F1	CTTCTAGATACCCGACTTTCCTTTTGCTCTG	5' region flanking *SacB* for mutagenesis of *XopN* in *Xoo*
XopN-R1	GCTTCAACATGATGCAGCAGGTTTCATCAC	
XopN-F2	CTGCTGCATCATGTTGAAGCGCTGTTGAGTC	3' region flanking *SacB* for mutagenesis of *XopN* in *Xoo*
XopN-R2	AAGTCGACAAGACCTTCAACAGCGGCTACG	
XopV-F1	ATGGATCCGGCACATTGAGAAACAACAC	5' region flanking *SacB* for mutagenesis of *XopV* in *Xoo*
XopV-R1	CCATTACTGGGGATGGGTGAGCTGAAAATG	
XopV-F2	CACCCATCCCCAGTAATGGCAGCTTGGTCATC	3' region flanking *SacB* for mutagenesis of *XopV* in *Xoo*
XopV-R2	ATCTGCAGACCGTTAGGGTCAGAATGTGC	
XopZ-F1	TAGGATCCACAGGATCATCGAATGCAC	5' region flanking *SacB* for mutagenesis of *XopZ* in *Xoo*
XopZ-R1	CGAAGAGTTGGATGGATGCGAGATTGTCTC	
XopZ-F2	CGCATCCATCCAACTCTTCGACCACCGAATC	3' region flanking *SacB* for mutagenesis of *XopZ* in *Xoo*
XopZ-R2	ACCTGCAGTCAGCGAGGCGAATTTGTTGTC	
flic-F	atgGGATCCATGGCACAAGTAATCAACACCAAC	PCR-amplification of fliC for preparation o f flagellin
flic-R	ccaaagcTTACTGCAGCAGGCTCAGCACGT	

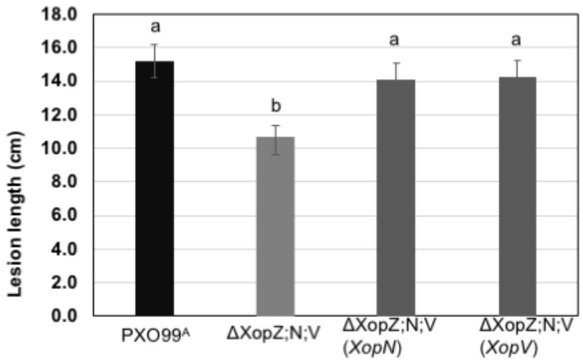

Figure S1. Complementation of triple mutant (ΔXopZ;N;V) with Non-TAL effectors XopN and XopV for pathogenicity in rice Kitaake. Measurements of lesion lengths caused by PXO99^{A} and its derivatives mutants as indicated below each column. The measurements each were obtained from 10 leaves of five rice plants of Kitaake (two leaves per plant). Values with the same lowercase letters above columns do not differ significantly at the <0.5 level using Tukey statistics and ANOVA analysis. Bars indicate standard deviation (SD).
